# Assessment of Soil Fertility Using Induced Fluorescence and Machine Learning

**DOI:** 10.3390/s22124644

**Published:** 2022-06-20

**Authors:** Louis Longchamps, Dipankar Mandal, Raj Khosla

**Affiliations:** 1 School of Integrative Plant Sciences, Cornell University, Ithaca, NY 14853, USA; 2 Department of Agronomy, Kansas State University, Manhattan, KS 66506, USA; dmandal@ksu.edu (D.M.); rkhosla@ksu.edu (R.K.); 3 Department of Soil & Crop Sciences, Colorado State University, Fort Collins, CO 80523, USA

**Keywords:** soil fertility, induced fluorescence, precision agriculture, proximal soil sensing

## Abstract

Techniques such as proximal soil sampling are investigated to increase the sampling density and hence the resolution at which nutrient prescription maps are developed. With the advent of a commercial mobile fluorescence sensor, this study assessed the potential of fluorescence to estimate soil chemical properties and fertilizer recommendations. This experiment was conducted over two years at nine sites on 168 soil samples and used random forest regression to estimate soil properties, fertility classes, and recommended N rates for maize production based on induced fluorescence of air-dried soil samples. Results showed that important soil properties such as soil organic matter, pH, and CEC can be estimated with a correlation of 0.74, 0.75, and 0.75, respectively. When attempting to predict fertility classes, this approach yielded an overall accuracy of 0.54, 0.78, and 0.69 for NO_3_-N, SOM, and Zn, respectively. The N rate recommendation for maize can be directly estimated by fluorescence readings of the soil with an overall accuracy of 0.78. These results suggest that induced fluorescence is a viable approach for assessing soil fertility. More research is required to transpose these laboratory-acquired soil analysis results to in situ readings successfully.

## 1. Introduction

To achieve a higher level of precision in nutrient management, farmers require information about soil fertility at every location of their fields. However, the acquisition and processing of soil samples is time consuming, labor intensive, and the associated cost remains unaffordable to farmers [1]. Moreover, soil samples acquired at a sampling density of one sample per ha (i.e., common commercial practice for field mapping) often fail to characterize the spatial variability of soil properties that happens at a much smaller scale [2]. Proximal soil sensing (PSS) can be used to estimate soil properties (<2 m below soil surface) rapidly at a high sampling density [3]. It can be in situ or mobile. For decades, agronomists and soil scientists have used color as a soil classification parameter [4]. Findings reported in more recent research studies suggest that soil’s optical properties are influenced by its physical and chemical properties [5]. Optical techniques were investigated using visible, near-infrared, and mid-infrared reflectance spectroscopy and were reported to be very good (R^2^ > 0.81) predictors of soil carbon and organic matter and good (R^2^ 0.61–0.81) predictors of soil texture, calcium (Ca), cation exchange capacity (CEC), and magnesium (Mg), among others [6]. Near-infrared reflectance can be used to detect soil organic matter content (r > 0.9) of dried soil samples [7]. This concept is widely used in the commercial optical soil sensors (OpticMapper; Veris Technologies Inc., Salina, KS, USA) that enable on-the-go detection of organic matter using visible and near-infrared wavebands. More recent sensing platforms can be used attached to another implement to characterize the soil while implementing another task. For instance, the Smart Firmer (Precision Planting, Tremont, TN, USA) is attached to the seeder’s firmer and uses optical sensors to estimate soil organic matter and moisture on-the-go while seeding the crop, and the Veris iScan (Veris Technologies Inc., Salina, KS, USA) is attached to the tillage tool, fertilizer bar, or planter to map soil organic matter, electrical conductivity, moisture, and temperature on-the-go. A new generation of soil sensors also uses soil optical properties to try to detect soil properties such as nitrogen (N), phosphorus (P), potassium (K), organic matter (OM), pH, moisture, CEC, and minor nutrients in situ and in real time (ChrysaLabs Inc., Montreal, QC, Canada; AgroCares Scanner, Wageningen, The Netherlands).

One aspect of optical soil sensing that has received low attention for the characterization of soil properties is ultra-violet (UV)-visible (Vis)-induced fluorescence [8]. Active fluorescence measurement has long been implemented in laboratory conditions, and mobile platforms were limited by both the power of the excitation energy source and the weakness of the fluorescence signal itself [9]. With recent technology developments, notably the advent of powerful UV light emitting diodes and increasingly sensitive optical sensors, portable fluorescence sensors can now be brought to the field. A review of PSS techniques did not mention the use of fluorescence to predict soil physical and chemical properties, most likely due to the scarcity of literature on this topic [3]. In a recent series of research experiments on the topic of soil characterization using portable fluorometers, it is reported that several chemical and physical soil properties such as texture, pH, and CEC can be detected using X-ray fluorescence [8,10,11,12]. More recently, a study assessed the potential of UV-induced fluorescence in the visible spectrum to assess soil properties and found that the predictive ability of UV-induced fluorescence was very significant for topsoil [13]. They suggested that this approach could be useful for farmers wanting to update information on soil fertility for fertilization purposes.

Induced fluorescence emitted by a material is influenced by both the material’s waveband-selective light absorption properties and its capacity and specificity to emit fluorescence [14]. Fluorophores are chemical compounds that have the property to emit fluorescence upon light absorption. Soil fluorophores can either be organic (present in humus as coumarins, quinones, 2-aminobenzoic acid, salicylic acid, among others) or inorganic [15]. Soil is a mixture of several organic and inorganic compounds that can potentially emit fluorescence. Martins et al. [16] reported the fluorescence spectrum of a whole soil sample as showing a broad (from 480 to 630 nm) spectrum with a peak around 520 nm. Consistently, [17] observed the laser induced fluorescence spectrum of a whole soil sample to be broad in the visible wavebands, and they also observed a much lower fluorescence intensity once the organic fraction of the sample was removed. Laser-induced fluorescence can be used to determine the humification of soil organic matter [16]. Portable X-ray fluorescence was used in situ to measure soil Ca, potassium (K), manganese (Mn), iron (Fe), copper (Cu), and other micro-elements to predict sand, silt, and clay content with success [8,18].

An optical sensor based on fluorescence allows for in situ measurement of UV-Vis-induced fluorescence, the Multiplex3 (Force-A, Orsay, France). This sensor was developed to measure anthocyanins and flavonoids in grapes and provide precise evaluation of fruit maturation [19]. This sensor was also shown to provide early estimates of N status in maize (*Zea mays* L.), rice (*Oryza sativa* L.), and potatoes (*Solanum tuberosum* L.), among other crops [20,21,22,23]. Studies also demonstrated the potential to detect biotic stresses such as powdery mildew in sugar beets (*Beta vulgaris* L.) and various fungal infections in wheat (*Triticum aestivum* L.) using this sensor [24,25]. This indicates that handheld UV-Vis-induced sensors can be versatile in their uses, providing an interesting tool to characterize crops for precision agriculture. The study conducted by [13] found encouraging results using UV-Vis-induced fluorescence to predict soil chemical properties of air-dried samples.

Fluorescence spectroscopy consists of measuring the photoluminescence of molecules that emit light after having absorbed ultraviolet, visible, or infrared light. For plants, pigments (chlorophyll and anthocyanin) act as key responsive parameters for fluorescence emission at a specific wavelength of excitation. Differential sensitivity of the emission spectrum (at far-red and red) at specific excitation wavelengths (green and red) can be observed in wheat leaves [26]. These differences in the emission spectrum help to rationalize the chlorophyll indices (SFR_G and SFR_R) and anthocyanin indices (ANTH_RG and ANTH_RB) for plants that are generated by the Multiplex3 sensor. The soil fluorescence excitation-emission matrix also demonstrates such differential sensitivities at the FRF and RF emission spectrum [27]. Among soil components, organic matter components such as humic and fulvic acids have fluorescent properties [28,29]. Such fluorescent behavior relies on the aromaticity, aliphatic character, degree of polycondensation, content of carboxylic groups or organic free radicals, or presence of amide groups or polysaccharidic structures’ pH value [7,13,30,31]. Such differences in the emission spectrum induced by excitation wavelengths can be potentially traced using similar indices such as SFR_G and SFR_R. Hence, it was hypothesized that soil parameters might be predicted via a fluorescence spectroscopy instrument designed for plants.

The hypothesis of this project was that UV-Vis-induced fluorescence sensors can be used for instantaneous assessment of soil fertility. The specific objectives were to assess if induced fluorescence of soil can be used to (1) estimate soil chemical properties, (2) classify soil samples within fertility classes of soil properties, and (3) predict the N fertilizer rate recommendation.

## 2. Materials and Methods

### 2.1. Sites and Soil Sampling

Field data were acquired during the crop growing season over two years (2013 and 2016) from nine sites located in Colorado (Table 1). Fields were selected with the information from the farmers about contrasting soils within their farm. The objective was to acquire soil samples having a broad range of values for each soil property. Soil was sampled from the top 20 cm at random locations within fields with a 2.5 cm diameter soil sampling probe. At each location, samples were composites collected to total about 500 g of wet soil (6 to 10 cores collected within a 1 m radius circle around the geolocated sampling point). Soil samples were air dried, evenly spread in a shallow container, and scanned with a fluorescence sensor (described below). After fluorescence reading acquisition, soil samples were sent to Servi-Tech Laboratories (Dodge City, KS, USA) where soil analysis was performed. Soil pH and soluble salts were determined by measurement in a 1:1 soil:water slurry. Soil organic matter content (SOM) was determined using the weight-loss-on-ignition method [32]. Soil NO_3_–N was determined by the Cd reduction method, and P was extracted by the Mehlich-3 method [33,34]. Soil concentration in K, S, Ca, Mg, and Na was measured with the ammonium acetate method [35]. Soil concentration in Zn, Fe, Mn, and Cu was measured with the diethylenetriaminepentaacetic acid (DTPA) method [36]. Particle size analysis (soil texture) was performed using the hydrometer method [37].

### 2.2. Fluorescence Sensor

**Figure 1:**
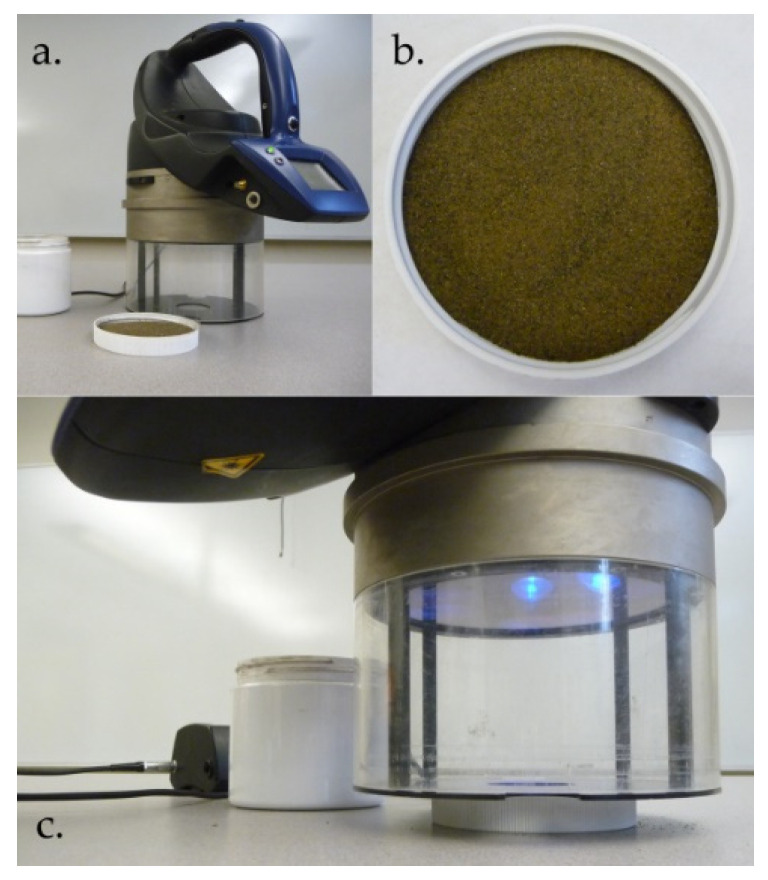
Multiplex MX3 sensor (**a**), soil disposed in a plate (container lid) ready for sensing (**b**), and fluorescence acquisition of the soil sample with the Multiplex MX3 (**c**).

The sensor used for this study was the portable Multiplex MX3 multi-parameter fluorescence sensor (FORCE-A, Orsay, France; Figure 1a). The four excitation channels are UV (around 375 nm), blue (around 470 nm), green (around 515 nm), and red (around 625 nm; Table 2). Excitation light pulses (20 μs per flash) are delivered by high-power light emitting diode arrays located around the detectors and pointing in the direction of the sensed area. The three detection channels are yellow (590 nm ± 40 nm; YF), red (678 nm ± 22 nm; RF), and far-red (750 nm ± 65 nm; FRF). The detectors consist of three silicon photodiodes (20 mm × 20 mm), each having an optical bandpass filter allowing only YF, RF, or FRF light to reach the photodiode. The flash induces the emission of fluorescence, and the filters allow the selection of the wavebands of interest. A firmware synchronizes the light pulses and the detectors in order to acquire each combination (12 in total) of excitation wavebands and detection channels for about 70 readings per second. Fluorescence based indices are generated by the sensor’s firmware (Table 3). More details about the sensor hardware can be found in Cerovic et al. [40].

### 2.3. Data Acquisition

Fluorescence readings were acquired in laboratory conditions on air dried soil samples. Dried soil samples were placed in a plate (container lid) to an even 1 cm thickness of soil (Figure 1b). A black metal mask was installed on the sensor to restrain the field-of-view of the sensor to a 40 mm diameter circle (Figure 1a). The Multiplex MX3 was placed on the soil plate and triggered to acquire the fluorescence measurement (Figure 1c). The sensor was set to acquire an average of over 250 induction/detection cycles for each sample. Soil measurements were acquired over the 168 soil samples collected from the field.

### 2.4. Statistical Analysis

Descriptive statistics including average, minimum, maximum, standard deviation, skewness, and kurtosis were calculated for soil properties over the 168 soil samples in this database. The R software [41] package stats was used to produce the descriptive statistics. Density plots were generated for the soil target variables to realize the underlying distribution of the data. Subsequently, the Anderson–Darling test was performed to determine whether the data followed a normal distribution. This type of test is useful for testing normality, which is a common assumption used in many statistical approaches including linear regression and ANOVA. The test statistics (TS) value of the Anderson–Darling test is generally compared to each critical value that corresponds to significance level α = 0.01 and 0.05 to see if the test results are significant. The density plots indicated non-normal distribution for all soil target variables (data not shown). The Anderson–Darling test statistics results were significant at both significance levels, which means the null hypothesis can be rejected. It was apparent from the test statistics that the sample data sets were not normally distributed.

#### 2.4.1. Estimation of Soil Properties with Random Forest Regression (RFR)

Linear regression techniques are often used for estimation of dependent parameters (soil properties) from independent parameters (optical measurements). However, the linear regression is a parametric method that needs explicit modeling of nonlinearities in the data and interactions between the parameters [42]. Moreover, the inferential procedures for linear regression are typically based on a normality assumption for the residuals. For estimation of soil properties, these conditions rarely apply since data distributions are usually not known a priori. Moreover, the optical measurements in soil fluorescence spectroscopy are not always independent of each other. In such conditions, machine learning-based methods such as Random Forest Regression (RFR) are more robust, as they do not require that the underlying distribution of the data be known a priori and do not assume independence amongst the predictors. The RFR is a nonparametric method, and unlike linear regression, it does not require nonlinearities and parameter interactions to be explicitly modeled since these can be learned from the data themselves.

In recent years, RFR has been used widely in applications related to soil-crop sensing methods [43,44,45]. The RFR is an ensemble learning technique developed by Breiman [46] that involves combining a large set of decision trees generated independently so that no two trees are the same. The independence between the trees is achieved by randomly selecting one third of the predictors at each node for node splitting and by using a random bootstrap sample comprising about 67% of the training samples to build each tree of the random forest. The remaining 33% of samples are called out-of-bag samples that are used to obtain an error estimate based on the bootstrap subset. At each node, the best split is chosen to form child nodes [47]. The value of each child node is the average of the sample values in that node. The node splitting is based on minimization of the Mean Squared Errors for a tree.

The RFR also identifies a subset of important independent parameters from the total parameter set which are relevant for the regression model for each independent parameter. In RFR, the most used approach is the Mean Decrease in Impurity (MDI) score-based feature importance. Features with high scores are further used in the regression model for each soil parameter. The Pearson’s *r* coefficient of correlation between observed and estimated values were calculated for the training and test datasets. The Random Forest regression model training and validation were implemented using the open-source Python Scikit-learn packages.

#### 2.4.2. Predicting Fertility Classes

To assess the potential of the Multiplex MX3 as a maize fertilization management tool, soil properties were classified as per the maize fertilization guide of the Colorado State University Extension [48,49] (Table 4). Three, four, or five classes were created depending on the soil property (Table 4). Soil properties that most often require intervention in Colorado soils (i.e., NO_3_-N, SOM, P, K, Zn, and S) were reported as well as salts’ and minor elements’ (i.e., Mn and Cu) content [48,50]. From these soil properties, only the ones for which there was enough representation in each fertility class were used for classification, notably NO_3_-N, SOM, and Zn.

The random forest classifier was used to predict the soil fertility classes based on fluorescence readings. Similar to the RFR approach, the random forest classifier uses an ensemble learning technique which is constructed by several decision trees that are trained, and their results are combined through a voting process by the majority of the individual decision trees [46]. The multiple decision trees of the random forest are trained on a bootstrapped sample of the original training data. In general, the random forest increases the diversity among the decision trees by randomly resampling the data with replacement and by randomly changing the parameter subsets for node splitting at each node of every decision tree. The R package “random Forest” was used to conduct the random forest classification [41,51].

From the random forest classification analysis output (i.e., soil fertility classes estimated from fluorescence readings), a confusion matrix was built between observed and estimated values, which enabled quantification of true and false positives and calculation of prediction performance statistics. For each class, the area under the curve (*AUC*) was calculated as follows:

(1) $$AUC=\frac{TP\times FP}{2}+\frac{\left(1-FP\right)\times \left(1+TP\right)}{2}$$

where *TP* is the true positive rate, and *FP* is the false positive rate. The *AUC* was computed for each class of each selected soil property. The *AUC* ranges from 0 to 1 with an *AUC* below 0.5 indicating estimation worse than randomness, an *AUC* of 0.5 being equivalent to random prediction, and an *AUC* of 0.6–0.7, 0.7–0.8, 0.8–0.9, or 0.9–1 indicating a poor, average, good, or excellent prediction potential, respectively. The overall accuracy (*OA*) and the balanced accuracy (*BA*) were calculated for both the training and the test dataset of each soil property.

The *OA* is calculated as follows:

(2) $$OA=\frac{\sum_{i=1}^{c}TP_{i}}{N}$$

where *OA* is the overall accuracy; *i* is the *i*th class (e.g., low, medium, high); *c* is the number of classes in the confusion matrix; *TP_i_* is the number of true positives for class *I*; and *N* is the total number of observations in the dataset.

The *BA* is calculated as follows:

(3) $$BA=\frac{\sum_{i=1}^{c}Recall_{i}}{c}$$

where *BA* is the balanced accuracy; *i* is the *i*th class (e.g., low, medium, high); *c* is the number of classes in the confusion matrix; and *Recall* is the number of correctly estimated observations in class *i* out of the number of actual observations in class *i* [52]. The *BA* cannot be calculated with classes containing zero observations, and thus classes with zero observation are eliminated before calculation of *BA*. The *BA* enables a better prediction for under-represented classes than the traditional overall accuracy, which provides a prediction assessment for the entire dataset. A simple measure of accuracy may be misleading and is better interpreted when accompanied by a baseline calculation. The baseline accuracy (BASE) is calculated by classifying all samples as the most common class in the dataset and by using equation 2 to measure the *OA* of this modified confusion matrix.

In order to compare the prediction of continuous data (Obj. 1) to the prediction of fertility classes (Obj. 2), the continuous data were converted to classes using Table 4. This enabled the comparison of both approaches based on overall accuracy rather than comparing Pearson’s *r* of the regression method to the overall accuracy of the classification method.

The *AUC*, BASE, *OA*, and *BA* were reported for each selected soil properties. A custom R code was written to compute the confusion matrix, the baseline, the *OA*, the *BA*, and the *AUC* [41].

#### 2.4.3. Predicting Nitrogen Rate Recommendation

The N rate recommendation for maize can be calculated based on the soil NO_3_-N and SOM content [53]. The decision algorithm is shown in Table 5. For each soil sample, the recommended N rate was calculated based on this decision algorithm. Subsequently, for each sampling point, the N rate is tabulated against corresponding fluorescence readings. A random forest classification model was generated using fluorescence readings as predictors and the N rate as the target variable. This random forest based trained model allowed us to estimate the N rate directly for a given fluorescence reading.

## 3. Results and Discussion

### 3.1. Statistical Description of Soil Properties

As anticipated, the collected soil samples from nine experimental sites displayed a wide range of values for most soil properties (Table 6). As per the Kurtosis of the distributions, most soil properties showed a fair level of variability, except for Zn and Cu, for which the standard deviation was low, and Kurtosis was high. The range of values (i.e., minimum to maximum) of NO_3_-N, SOM, P, and Zn included all levels of soil fertility (Table 4), and the range of values of S and Salts included at least two levels of soil fertility from Table 4. The soil properties NO_3_-N, SOM, and Zn had at least 10% of observations in each fertility class, while the soil properties P and Fe had less than 10% of observations in the low classes. Other soil properties that could be divided into fertility classes as per Table 4 (i.e., K, S, Salt, Mn, and Cu) had classes with 1% or fewer observations. This indicates that NO_3_-N, SOM, and Zn properties did provide enough data to meet the objective of assessing classification of fertility based on fluorescence data, while P and Fe may provide only partial insights, and the other properties are not suitable to meet this objective.

### 3.2. Fluorescence Features to Estimate Soil Parameter Using RFR

The details of the RFR feature importance are presented in Table 7. Results showed a strong disparity in terms of which optical feature was the most important for each soil parameter based on MDI scores (Table 7). When considering only the twelve signals, and not the optical ratios and parameters, YF_R, YF_UV, and YF_G were the most frequent important predictors. This indicates that the yellow filter seems important for soil properties’ prediction. As mentioned in the previous section, YF_R and YF_G are reflectance rather than fluorescence signals (Table 2) because induction wavebands for these signals cannot trigger fluorescence in the yellow part of the spectrum. Nevertheless, because YF_UV is also one of the best predictors, it does not lead to concluding that reflectance is better than fluorescence to estimate soil properties. The yellow filter (~590 nm) signals being better predictors opens the question of the performance of filters for shorter wavebands such as the blue-green filter, which is an option with the Multiplex MX3 system. The UV-induced fluorescence (351 nm) of whole soil samples shows the highest intensity of fluorescence from 475 to 525 nm, which may be an optimal zone for detecting subtle differences in soil samples’ fluorescence [17]. Better performances with filters in shorter wavebands are consistent with [54] who observed greater disparity in fluorescence intensity from soils’ humic acid component at shorter (~400 to 600 nm) wavebands. In their study, the greatest differences in fluorescence from soils coming from different sampling locations appeared around 475 nm. In the current study, the yellow filters performed better as independent signals, and the literature seems to suggest that shorter wavebands’ filter (e.g., blue) may perform even better.

In terms of induction channels, the fluorescence measurements with UV and red induction were more important in predicting soil properties as per MDI scores (Table 7), followed by green and blue induction bands. Even if fluorophores react differently to different induction bands, it is unclear why this sequence was observed because few studies have tested the effect of using different induction bands on the discrimination potential of whole soil samples. Although conducted on forest soil from the Amazon Forest with high organic matter content, a study using two different induction wavebands (i.e., 378 nm and 445 nm) observed resulting fluorescence spectra with marked differences, notably with two peaks for the shorter waveband and a single peak for the longer waveband [55]. This indicates that different fluorophores may be triggered with different induction wavebands, thus resulting in different fluorescence spectra. In this study, both red and UV induction bands performed somewhat similarly in terms of prediction and may be preferred to other bands for designing a soil properties sensor that contains fewer elements (e.g., number of induction LEDs).

In general, ratios and fluorescence indices provided better prediction power than individual signals (Table 7). For instance, when cumulating relative importance (i.e., MDI scores) of each signal over all soil properties in Table 7, the two best indices, NBI_UVm and FLAV, were 5.4 and 2.6 times more important, respectively, than the best predictor signal (YF_R) for estimating soil properties. Both indices use signals with UV and/or red induction, which are the induction bands that provided the best prediction potential as per results shown above. However, no indices from Table 3 used the YF filter signals, which were the best raw signals to predict soil properties as per results shown above. This is related to the Multiplex MX3 being originally designed to sense fruits and vegetation rather than soil, and all proposed indices target vegetation fluorophores such as chlorophyll, anthocyanins, and flavonoids [40]. For instance, the NBI_UVm is a fluorescence index that is not automatically generated by the sensor, and yet, it is the one that yielded the best results for the prediction of soil properties. As suggested by Longchamps and Khosla (2014), it is possible that the calculation of the index using averaged signal values over 250 readings, rather than calculating the index 250 times and then averaging it, may have helped to stabilize the values and improved the estimation power. Findings from the current study suggest that research should be conducted on full spectrum fluorescence of whole soil samples induced by different wavelengths to identify new indices dedicated to soil analysis.

### 3.3. Estimating Soil Parameters Using Induced Fluorescence

**Figure 2:**
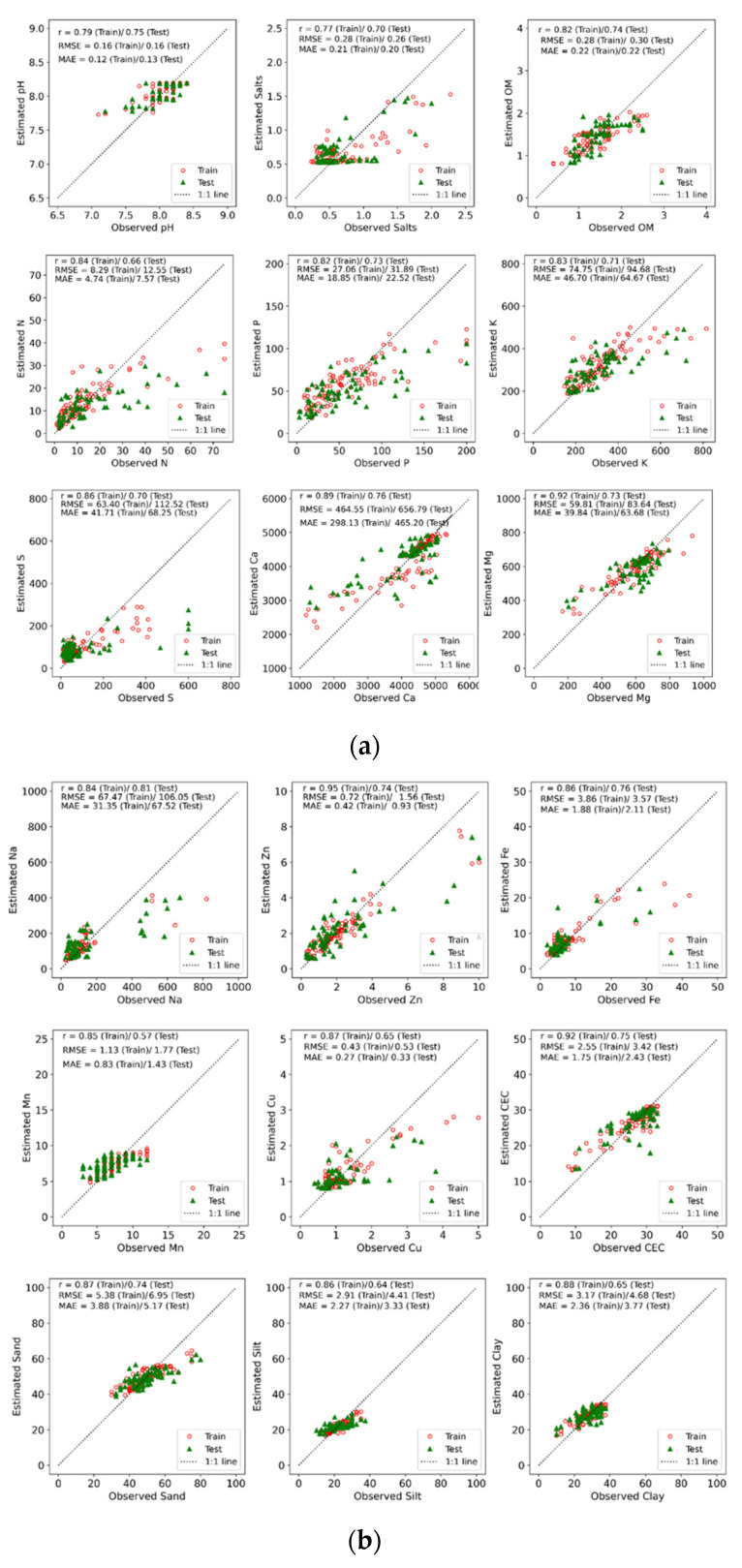
(**a**) Scatter plot of the estimated to observed values of each soil property as per random forest regression analysis. The Pearson’s *r* coefficient of correlations and two error estimates, i.e., Root Mean Square Error (RMSE) and Mean Absolute Error (MAE), are indicated for both training and test dataset for each soil property. (**b**) Scatter plot of the estimated to observed values of each soil property as per random forest regression analysis. The Pearson’s *r* coefficient of correlations and two error estimates, i.e., Root Mean Square Error (RMSE) and Mean Absolute Error (MAE), are indicated for both training and test dataset for each soil property.

The Figure 2a,b show scatter plots between the observed and estimated values of the training and test datasets for each soil properties and report the Pearson’s *r* coefficient of correlation. In general, there was a large difference (i.e., average of 0.15-point difference) between the *r* values of the training versus the test datasets, which seems to indicate a slight underfitting of the training data associated with a small number of observations covering a limited range of values. Nevertheless, RFR is an artificial intelligence technique that is less prone to under/overfitting, and thus due diligence was conducted to avoid overfitting [46].

When considering the Pearson’s *r* coefficient of correlation for the test dataset, all soil properties displayed an *r* value of 0.57 or above between the estimated and observed values (Figure 2a,b). This demonstrates that there was a significant positive correlation when trying to predict soil properties using optical features, which was one of the main objectives of this study. Nevertheless, the *r* values ranged from 0.57 for Mg to 0.81 for Na, indicating that this approach did not perform similarly across all soil properties. There was no discernible commonality among the soil properties with the highest *r* values, and neither among the soil properties with the lowest *r* values. Nevertheless, soil organic matter (SOM) is a soil fraction containing several fluorophores [31,56], and it did appear among the top five soil properties with an *r* value of 0.74. Ref. [57] observed a drastic difference between the UV-induced fluorescence spectrum of the whole soil sample with and without (i.e., calcinated soil) the organic matter. This indicates that the large majority of whole soil sample fluorescence is emitted from organic matter, which explains the relatively high *r* value (i.e., 0.74 for the test dataset) between observed and estimated SOM in this study. Other soil properties, notably pH, CEC, Fe, Ca, and Na, showed a better correlation between observed and estimated values than SOM with Pearson’s *r* of 0.75, 0.75, 0.76, 0.76, and 0.81, respectively, for the test dataset (Figure 2a,b). Ref. [13], who used the Multiplex MX3 equipped with a blue-green filter in place of the yellow filter to estimate chemical properties of whole soil samples, also found pH, Ca, CEC, and Fe on top of the list in terms of predictability. In their study, the Na prediction using the same system showed lower accuracy. Interestingly, pH and CEC are soil properties rather than elemental concentrations, and yet both showed high potential for predictability using UV-induced fluorescence. Because of their influence on soil fertility and elemental concentration, other soil properties combined can act as a proxy for determining soil pH (e.g., Al, Fe, and Mn) and CEC (e.g., Ca, Mg, K, Cu, Zn, and Fe) using induced fluorescence [11,58]. This may explain the higher accuracy of induced fluorescence to predict pH and CEC.

Overall, the simple use of the portable Multiplex MX3 sensor enabled high prediction of a number of soil properties (e.g., SOM, pH, and CEC) that are important for assessing soil fertility. This indicates that, as also observed by [13] and by [11,12], fluorescence sensing has good potential for the rapid assessment of soil fertility. As indicated by the observed performance and statistics of the current dataset, a larger dataset showing a wider range of each soil property is required to improve the prediction algorithms further. While obtaining a precise estimate of the soil properties is desirable from an agronomic standpoint, categorical estimates may be more convenient for decision making while requiring less accurate sensing technologies.

### 3.4. Estimating Fertility Classes of Selected Soil Properties Using UV-Induced Fluorescence

The soil properties NO_3_-N, SOM, and Zn had enough observations (*n* ≥ 10%) in each fertility class to test the second objective (i.e., predicting fertility classes with fluorescence) of this study. Results show that the *OA* was 0.54, 0.78, and 0.69 for the test dataset of NO_3_-N, SOM, and Zn, respectively (Table 8). This represents an improvement of 0.23, 0.12, and 0.25 points over the baseline accuracy for NO_3_-N, SOM, and Zn, respectively. In general, the *BA* was slightly inferior than the *OA*, indicating an unbalanced distribution of observations across the classes. The hypothesis for this procedure was that trying to predict broad classes would yield better results than trying to predict exact values, with the rationale that, in the end, exact values are often converted to fertility classes for decision making. These results seem to suggest that this approach did not yield substantial improvement over a direct regression approach. Because of the inability directly to compare regression performance coefficients such as Pearson’s *r* to classification performance coefficients such as *OA*, continuous data output from the RFR was converted to classes. The *OA* of the continuous data output from RFR converted to classes using Table 4 was 0.50, 0.75, and 0.60 for the test dataset of NO_3_-N, SOM, and Zn, respectively (data not shown). The results from the class prediction method thus suggest a slight improvement (i.e., 0.05-, 0.03-, and 0.09-point improvement for NO_3_-N, SOM, and Zn, respectively) as compared to the prediction of continuous values. Since predicting classes is not an additional burden for computation as compared to predicting continuous values and that continuous values are often converted to classes, these results suggest that predicting classes should be the preferred option. Trontelj ml. and Chambers [59] observed an improvement in prediction results with a decrease in the number of classes when using artificial intelligence to predict soil properties out of the optical spectral signature. Although hardly comparable across different properties, the results of the current study seem to indicate that a larger number of classes (e.g., five classes for NO_3_-N) yielded lower *OA* as compared to fewer classes (e.g., three classes for SOM and Zn). Overall, the class prediction approach seems to be preferable over the continuous prediction approach, and there is a trend towards a fewer number of classes yielding better prediction accuracy.

### 3.5. Estimating N fertilization Recommendation Directly Using UV-induced Fluorescence

The fluorescence features were used to measure the N rate recommendation of each sample directly as it was calculated using the algorithm in Table 5. Among all possible cases in the algorithms, only the N rates 0, 39, 95, 151, 179, 207, and 235 kg N Ha^−1^ were represented. The confusion matrix was thus generated only with those seven classes. The baseline accuracy (BASE) was 0.41 and 0.28 for the training and the test datasets, respectively. Using machine learning to predict the N recommendation rates, the *OA* was 0.91 and 0.78 for the training and test datasets, respectively (Table 9). This represents an improvement of 0.5 points for both the training and test datasets. The *BA* yielded similar results, suggesting a good distribution of cases in each class. These results seem to indicate that directly using UV-induced fluorescence measurements to predict the N fertilizer recommendation is a viable approach. There was no significant bias in terms of over- and under-prediction for both the training and the testing datasets. However, in some instances, the error of prediction was large (data not shown). For example, in the test dataset, one sample for which the N recommendation should have been 207 kg N per Ha was estimated at 0 kg N per Ha based on fluorescence measurements. Inversely, one sample for which the N recommendation should have been 39 kg N per Ha was estimated at 207 kg N per Ha. This indicates that despite a good overall accuracy (i.e., *OA* = 0.78 for the test dataset), important errors may occur, which could cause N loadings in the environment or yield loss if not addressed. Interestingly, there was no example in the literature of studies attempting to estimate fertilizer rates directly using soil optical properties. In the current study, this approach was attempted to make soil fluorescence sensing more practical for decision making. This was motivated by the availability of machine learning analytics enabling to account for a large number of factors for accurate prediction, and by the availability of the decision algorithm for the N rate based on soil properties (i.e., NO_3_-N and SOM). If this approach was to be implemented commercially, a different algorithm would be required for each different crop and possibly for different yield goals. Similar to other conclusions in this study, a larger number of samples would help confirm the potential of the approach, develop more robust algorithms, and provide a better assessment of the bias along with its magnitude. Nevertheless, this study clearly demonstrates the potential of this approach, and further development would inevitably require training the machine learning algorithm on a much larger dataset.

### 3.6. General Discussion

The dataset used in this study enabled the assessment of the potential of UV- and Vis-induced fluorescence to predict soil properties. Despite using a dataset of 168 samples, results showed that more data may be required to (1) train the machine learning algorithms and (2) provide a broader range of values for each soil property. The observations-to-features ratio of the training dataset was 4:1 (i.e., 100 observations to 25 features), which may be considered low as compared to the general rule of thumb of using a 10:1 ratio as a starting point [60]. However, random forest models are usually well suited for classification algorithms of small datasets, and previous studies show that prediction accuracy may not be impacted by the observations-to-features ratio as much as other machine learning approaches [61,62]. Nevertheless, in the case of the current study, despite using an adapted machine learning approach, there may not have been enough variability in the dataset for certain soil properties. For instance, the range of K, Mn, and Cu was all enclosed in one fertility class of Table 4. The results of this study thus do not allow to conclude for those soil properties and suggest that more samples may be needed to test the approach on a broader range of values.

As per the results of this study, the prediction of soil properties using induced fluorescence on whole dried soil samples presents a good potential for a rapid assessment of soil fertility, and possibly for fertilizer recommendations. Such an approach has multiple advantages over a traditional laboratory analysis and may even be transformational in countries where soil analysis laboratories are not available [63]. Yet, certain aspects are important to consider, one of which is the condition of the soil samples. It has been demonstrated that the presence of organic material (e.g., crop residues) in the sample may have a significant impact on the UV- and Vis-induced fluorescence readings [22]. It is thus important that samples be cleaned of such crop residues before the fluorescence readings’ acquisition. The moisture content of the sample may also impact the outcome of the readings. The effect of water on optical properties of soil is visible to the naked eye with wet samples appearing darker than dry samples, and this alteration of optical properties may also apply to fluorescence. Ref. [64] found a significant effect of soil moisture on trace element prediction by x-ray fluorescence, and the impact depended on the trace element measured and on the level of moisture content. This indicates that a soil wetness calibration curve may be required for each element independently. The absorption of light by liquid water in the UV to visible spectrum decreases down to about 420 nm and increases again after this minimum [65]. This indicates that UV induction may be more impacted by moisture than the visible light induction. In situ readings would present a significant advantage in terms of efficiency and practicality. However, the presence of crop residues and organic tissues on the surface as well as the variable levels of moisture in the soil can complicate the use of this technology. For instance, rather than a simple reading such as what would be used on the crop canopy, acquiring a reliable soil reading may require a cleaning of the surface from residues and the acquisition of separate soil moisture readings.

## 4. Conclusions

This study aimed at assessing the potential of a multi-parameter fluorescence sensor (Multiplex MX3) to predict soil properties of whole air-dried samples. Results showed that this approach can predict NO_3_-N, SOM, and Zn fertility classes with an overall accuracy of 0.54, 0.78, and 0.69, respectively. Certain fluorescence signals such as the ones using a yellow filter generated better results which seem to indicate that fluorescence at shorter wavebands may perform better than the longer ones for soil properties’ prediction. Moreover, the index NBI_UVm, which was calculated a posteriori, was the best index for soil fertility classification. This study also demonstrated that induced fluorescence can be used to predict the N rate directly (*OA* of 0.78), which indicates that this approach can be practical for farmers. Further development of this approach is required to explore its full potential and its expansion to in situ measurements. Notably, a larger training dataset composed of observations covering a broader range of soil properties’ values are required to confirm the potential for soil properties beyond NO_3_-N, SOM, and Zn, and to develop the random forest models further. Moreover, the transposition of this approach to in situ readings will require dedicated studies on the effects of organic components in the sensor field of view and on the effect of soil water content on the predictability of each soil property.

## Figures and Tables

**Table 1:** Location and sample size acquired along with soil classification for each site.

Site	Location (Lat. Lon.)	Sample Size	Soil Series ^†^
Site 1	Wellington, CO (40°40′ N, 104°59′ W)	60	Kim loam (Fine-loamy, mixed, active, calcareous, mesic Ustic Torriorthents)
		22	Nunn clay loam (Fine, smectitic, mesic Aridic Argiustolls)
Site 2	Atwood, CO (40°33′ N, 103°16′ W)	10	Nunn clay loam (Fine, smectitic, mesic Aridic Argiustolls)
		2	Haverson loam (Fine-loamy, mixed, superactive, calcareous, mesic Aridic Ustifluvents)
Site 3	Ault, CO (40°34′ N, 104°43′ W)	12	Kim loam (Fine-loamy, mixed, active, calcareous, mesic Ustic Torriorthents)
Site 4	Iliff, CO (40°46′ N, 103°02′ W)	8	Loveland clay loam (Fine-loamy over sandy or sandy-skeletal, mixed, superactive, calcareous, mesic Fluvaquentic Endoaquolls)
		6	Nunn clay loam (Fine, smectitic, mesic Aridic Argiustolls)
Site 5	Fort Collins, CO (40°36′ N, 104°59′ W)	6	Nunn clay loam (Fine, smectitic, mesic Aridic Argiustolls)
		4	Santana loam (Loamy, mixed, superactive, mesic Aridic Lithic Haplustolls)
Site 6	Severance, CO (40°31′ N, 104°52′ W)	10	Kim loam (Fine-loamy, mixed, active, calcareous, mesic Ustic Torriorthents)
Site 7	Lucerne, CO (40°28′ N, 104°41′ W)	5	Colby loam (Fine-silty, mixed, superactive, calcareous, mesic Aridic Ustorthent)
		4	Weld loam (Fine-silty, mixed, mesic, Aridic Argiustoll)
		1	Ascalon loam (Fine-loamy, mixed, mesic, Aridic Argiustoll)
Site 8	LaSalle, CO (40°17′ N, 104°39′ W)	5	Olney fine sandy loam (Fine-loamy, mixed Ustolic Haplargids)
		6	Otero sandy loam (Coarse-loamy, mixed (calcareous), mesic Ustic Torriorthents)
Site 9	Pierce, CO (40°36′ N, 104°42′ W)	5	Docono clay loam (Clayey over sandy or sandy-skeletal, smectitic, mesic Aridic Argiustolls)
		2	Nunn clay loam (Fine, smectitic, mesic Aridic Argiustolls)

^†^ [38,39].

**Table 2:** Presentation of the nine fluorescence and two reflectance (underlined) signals acquired by the Multiplex MX3 at each reading. Subscripts indicate the induction channel.

**Detection** **Channel**		**Induction Channel**			
		**UV**	**Red (R)**	**Green (G)**	**Blue (B)**
	Yellow (YF)	*YF_UV_*	*YF_R_*	*YF_G_*	*YF_B_*
	Red (RF)	*RF_UV_*	*RF_R_*	*RF_G_*	*RF_B_*
	Far-red (FRF)	*FRF_UV_*	*FRF_R_*	*FRF_G_*	*FRF_B_*

**Table 3:** Fluorescence indices used for this study along with their description and formula.

Parameter	Description	Formula *
SFR_G	Chlorophyll index with green induction	$\frac{1}{250}\sum_{i=1}^{250}\frac{FRF_{G_{i}}}{RF_{G_{i}}}$
SFR_R	Chlorophyll index with red induction	$\frac{1}{250}\sum_{i=1}^{250}\frac{FRF_{R_{i}}}{RF_{R_{i}}}$
FLAV	Index of compounds which absorbs at 375 nm, often flavonoids	$log\left(\frac{1}{250}\sum_{i=1}^{250}\frac{FRF_{R_{i}}}{FRF_{UV_{i}}}\right)$
FER_RG	Chlorophyll ratio originally designed for fruit anthocyanin content	$\frac{1}{250}\sum_{i=1}^{250}\frac{FRF_{R_{i}}}{FRF_{G_{i}}}$
FERARI	Index of anthocyanins on grapes	$log\left(\frac{1}{250}\sum_{i=1}^{250}\frac{5000}{FRF_{R_{i}}}\right)$
ANTH_RG	Index of anthocyanin with green induced denominator	$log\left(\frac{1}{250}\sum_{i=1}^{250}\frac{FRF_{R_{i}}}{FRF_{G_{i}}}\right)$
ANTH_RB	Index of anthocyanin with blue induced denominator	$log\left(\frac{1}{250}\sum_{i=1}^{250}\frac{FRF_{R_{i}}}{FRF_{B_{i}}}\right)$
NBI_R	Nitrogen balance index (red)	$\frac{1}{250}\sum_{i=1}^{250}\frac{FRF_{UV_{i}}}{RF_{R_{i}}}$
NBI_G	Nitrogen balance index (green)	$\frac{1}{250}\sum_{i=1}^{250}\frac{FRF_{UV_{i}}}{RF_{G_{i}}}$
NBI_Rm **	Ratio of UV induced far-red fluorescence on red light induced red fluorescence	$\frac{1}{250}\sum_{i=1}^{250}FRF_{UV_{i}}/\frac{1}{250}\sum_{i=1}^{250}RF_{R_{i}}$
NBI_Gm **	Ratio of UV induced far-red fluorescence on green light induced red fluorescence	$\frac{1}{250}\sum_{i=1}^{250}FRF_{UV_{i}}/\frac{1}{250}\sum_{i=1}^{250}RF_{G_{i}}$
NBI_Bm **	Ratio of UV induced far-red fluorescence on blue light induced red fluorescence	$\frac{1}{250}\sum_{i=1}^{250}FRF_{UV_{i}}/\frac{1}{250}\sum_{i=1}^{250}RF_{B_{i}}$
NBI_UVm **	Ratio of UV induced far-red fluorescence on UV induced red fluorescence	$\frac{1}{250}\sum_{i=1}^{250}FRF_{UV_{i}}/\frac{1}{250}\sum_{i=1}^{250}RF_{UV_{i}}$

* Induction waveband is in subscript. UV = Ultra-violet; G = Green; R = Red; B = Blue. ** This parameter was not automatically computed by the sensor but calculated afterward.

**Table 4:** Classification of selected soil properties values for maize fertilization in Colorado.

Soil Property			Soil Fertility Level		
	Very Low	Low	Medium	High	Very High
NO_3_-N (ppm)	0–6	7–12	13–18	19–24	>24
SOM (%)	-	0–1.0	1.1–2.0	>2.0	-
P (ppm) ^†^	-	0–10	11–31	31–56	>56
K (ppm)	-	0–60	61–120	>120	-
Zn (ppm)	-	0–0.9	1.0–1.5	>1.5	-
S (ppm)	-	0–6	6–8	>8	-
Fe (ppm) ^‡^	-	0–3	3–5	>5	-
Salts ^‡^	-	0–2	2–4	4–8	>8
Mn (ppm) ^‡^	-	0–0.5	>0.5	-	-
Cu (ppm) ^‡^	-	0–0.2	>0.2	-	-

^†^ P was not reported based on Melich-3 method in Davis and Westfall, 2014, but was reported by Bauder et al., 2003 [49], ^‡^ Classes for these properties come from Self, 2010 [50].

**Table 5:** Suggested nitrogen rates (kg N ha^−1^) for irrigated maize, as related to NO_3_-N in the soil and soil organic matter content, calculated from the algorithm. Target yield for this algorithm is 11 Mg grain per ha, and recommended N rate does not account for other N credits. Adapted from Davis and Westfall (2014) [48].

NO_3_-N (mg/kg) *	Soil Organic Matter (%)		
	0–1.0	1.1–2.0	>2.0
0–6	235	207	185
7–12	179	151	129
13–18	123	95	73
19–24	67	39	17
>24	11	0	0

* Average weighted concentration (mg kg^−1^) in the tillage and subsoil layers.

**Table 6:** Descriptive statistics of soil properties for the entire dataset.

	Mean	Min.	Max.	Standard Deviation	Kurtosis	Skewness
pH	8.09	7.10	8.40	0.24	3.31	−1.63
Salts	0.66	0.23	2.28	0.39	3.18	1.90
SOM ^†^ (%)	1.43	0.40	2.60	0.45	−0.05	0.43
NO_3_-N (mg/kg)	15	1	100	16	9.46	2.76
P (mg/kg)	59	3	284	49	6.13	2.13
K (mg/kg)	317	147	815	129	2.13	1.39
S (mg/kg)	97	7	709	124	7.64	2.64
Ca (mg/kg)	4113	1188	5322	950	1.68	−1.58
Mg (mg/kg)	594	167	931	125	2.34	−1.24
Na (mg/kg)	125	25	819	136	8.36	2.96
Zn (mg/kg)	2.2	0.3	20.8	2.4	21.84	3.99
Fe (mg/kg)	7.5	2.0	42.0	6.3	11.45	3.27
Mn (mg/kg)	7.1	3.0	23.0	2.5	10.85	2.30
Cu (mg/kg)	1.2	0.4	8.5	0.9	28.49	4.43
CEC ^‡^	26.9	8.0	33.0	5.51	2.55	−1.75
Sand (%)	49.3	30.0	80.0	9.34	1.09	0.83
Silt (%)	22.0	10.0	37.5	5.15	0.16	0.38
Clay (%)	28.8	10.0	37.5	6.11	1.01	−1.07

^†^ Soil organic matter content., ^‡^ Cation exchange capacity.

**Table 7:** Random Forest Regression feature or optical measurement importance for each soil parameter. The Mean Decrease in Impurity (MDI) scores are indicated along with a grayscale gradient showing higher values in darker gray tones. The sum of each column is equal to one.

Fluorescence Measurements	Soil Properties																	
	pH	Salt	OM	N	P	K	S	Ca	Mg	Na	Zn	Fe	Mn	Cu	CEC	Sand	Silt	Clay
YF_UV	0.00	0.01	0.00	0.05	0.13	0.03	0.01	0.17	0.01	0.01	0.01	0.01	0.26	0.08	0.02	0.01	0.02	0.02
RF_UV	0.00	0.00	0.01	0.02	0.04	0.01	0.03	0.04	0.01	0.00	0.01	0.01	0.05	0.00	0.09	0.01	0.02	0.00
FRF_UV	0.00	0.00	0.01	0.01	0.04	0.02	0.01	0.01	0.01	0.02	0.01	0.01	0.02	0.00	0.05	0.01	0.02	0.01
YF_B	0.01	0.01	0.02	0.01	0.02	0.01	0.05	0.20	0.01	0.00	0.01	0.01	0.03	0.02	0.04	0.02	0.02	0.02
RF_B	0.00	0.00	0.01	0.02	0.01	0.00	0.02	0.01	0.00	0.01	0.00	0.01	0.01	0.01	0.00	0.01	0.01	0.01
FRF_B	0.00	0.00	0.00	0.01	0.01	0.01	0.02	0.00	0.01	0.00	0.00	0.01	0.01	0.01	0.01	0.01	0.01	0.01
YF_G	0.01	0.01	0.30	0.02	0.19	0.06	0.02	0.02	0.01	0.00	0.02	0.01	0.06	0.04	0.02	0.03	0.01	0.01
RF_G	0.01	0.00	0.00	0.01	0.01	0.00	0.03	0.01	0.01	0.00	0.00	0.01	0.01	0.02	0.01	0.01	0.01	0.01
FRF_G	0.00	0.00	0.02	0.01	0.01	0.00	0.00	0.00	0.01	0.00	0.00	0.01	0.01	0.00	0.01	0.01	0.01	0.01
YF_R	0.10	0.00	0.41	0.01	0.08	0.03	0.02	0.04	0.01	0.00	0.03	0.03	0.04	0.04	0.03	0.03	0.02	0.01
RF_R	0.00	0.01	0.03	0.04	0.04	0.04	0.01	0.01	0.01	0.00	0.00	0.02	0.02	0.01	0.00	0.03	0.01	0.01
FRF_R	0.00	0.00	0.00	0.00	0.01	0.00	0.00	0.01	0.01	0.00	0.00	0.01	0.01	0.00	0.00	0.01	0.02	0.00
SFR_G	0.14	0.00	0.00	0.01	0.01	0.00	0.01	0.00	0.01	0.01	0.00	0.07	0.06	0.00	0.00	0.01	0.01	0.00
SFR_R	0.00	0.14	0.01	0.10	0.04	0.02	0.04	0.02	0.02	0.00	0.05	0.01	0.05	0.00	0.01	0.17	0.46	0.09
FLAV	0.08	0.59	0.00	0.20	0.01	0.45	0.39	0.06	0.10	0.26	0.01	0.01	0.02	0.01	0.04	0.04	0.02	0.14
FER_RG	0.01	0.00	0.00	0.04	0.02	0.05	0.00	0.01	0.02	0.00	0.01	0.03	0.02	0.00	0.01	0.02	0.02	0.03
ANTH_RG	0.01	0.00	0.00	0.03	0.02	0.05	0.01	0.01	0.02	0.01	0.01	0.03	0.04	0.00	0.00	0.02	0.02	0.06
ANTH_RB	0.02	0.00	0.01	0.16	0.01	0.02	0.01	0.01	0.03	0.01	0.01	0.01	0.04	0.01	0.02	0.03	0.03	0.10
NBI_G	0.04	0.00	0.00	0.01	0.01	0.00	0.01	0.01	0.01	0.00	0.00	0.03	0.04	0.01	0.01	0.01	0.01	0.00
NBI_R	0.00	0.09	0.01	0.12	0.06	0.08	0.10	0.02	0.02	0.22	0.01	0.01	0.02	0.01	0.02	0.08	0.02	0.02
FERARI	0.00	0.00	0.00	0.01	0.01	0.00	0.00	0.00	0.01	0.00	0.00	0.00	0.01	0.01	0.00	0.01	0.02	0.00
NBI_Rm	0.00	0.10	0.01	0.09	0.04	0.05	0.10	0.01	0.02	0.29	0.01	0.00	0.02	0.02	0.02	0.14	0.02	0.02
NBI_Gm	0.05	0.00	0.00	0.02	0.02	0.01	0.01	0.02	0.01	0.00	0.00	0.04	0.01	0.02	0.01	0.02	0.01	0.01
NBI_Bm	0.02	0.03	0.12	0.01	0.02	0.01	0.08	0.02	0.02	0.12	0.01	0.01	0.04	0.08	0.03	0.04	0.02	0.02
NBI_UVm	0.47	0.01	0.01	0.02	0.15	0.03	0.02	0.30	0.58	0.01	0.79	0.63	0.06	0.59	0.55	0.24	0.16	0.38

**Table 8:** Area under the curve (*AUC*) values for each class of each soil property that can be separated into fertility classes (see Table 4). The percentage of N (number of observation) in each class within each dataset is indicated within parenthesis. The baseline (BASE), overall accuracy (*OA*), and balanced accuracy (*BA*) calculated with the confusion matrix of each soil property are indicated.

	Training Dataset (*n* = 100)								Test Dataset (*n* = 68)							
Soil Parameter	Fertility Classes					BASE	*OA*	*BA*	Fertility Classes					BASE	*OA*	*BA*
	Very Low	Low	Medium	High	Very High				Very Low	Low	Medium	High	Very High			
NO3-N	0.82 (31)	0.71 (39)	0.66 (10)	0.75 (7)	0.92 (13)	0.39	0.65	0.68	0.81 (31)	0.72 (25)	0.66 (16)	0.54 (13)	0.74 (15)	0.31	0.54	0.50
SOM		0.87 (23)	0.79 (66)	0.68 (11)		0.66	0.84	0.81		0.86 (24)	0.72 (66)	0.50 (10)		0.66	0.78	0.57
P		0.83 (8)	0.84 (31)	0.77 (31)	0.90 (30)	0.43	0.80	0.66		0.50 (2)	0.79 (40)	0.63 (4)	0.78 (54)	0.40	0.66	0.48
K				1.00 (100)		1.00	1.00	1.00				1.00 (100)		1.00	1.00	1.00
Zn		0.82 (22)	0.60 (25)	0.81 (53)		0.53	0.74	0.70		0.80 (26)	0.60 (29)	0.79 (54)		0.44	0.69	0.64
S			0.50 (98)	0.50 (2)		1.00	0.98	1.00				1.00 (100)		1.00	1.00	1.00
Fe		0.64 (7)	0.54 (36)	0.65 (57)		0.50	0.65	0.90		0.50 (3)	0.55 (41)	0.57 (56)		0.56	0.60	0.37
Salt		0.50 (99)	0.50 (1)			0.99	0.99	1.00		1.00 (100)				1.00	1.00	1.00
Mn			1.00 (100)			1.00	1.00	1.00			1.00 (100)			1.00	1.00	1.00
Cu			1.00 (100)			1.00	1.00	1.00			1.00 (100)			1.00	1.00	1.00

**Table 9:** Accuracy of N rate prediction using fluorescence features. The baseline accuracy (BASE), overall accuracy (*OA*), and balanced accuracy (*BA*) were calculated from a multi-class confusion matrix. The percentage of cases when estimated N rate was below or above the actual recommended rate is indicated in the under- and over-estimated columns.

	N	BASE	*OA*	*BA*	Under-Estimated	Over-Estimated
Training	100	0.41	0.91	0.91	5%	4%
Test	68	0.28	0.78	0.77	10%	12%

## Data Availability

The data presented in this study are available on request from the corresponding author.
